# GEN-RWD Sandbox: bridging the gap between hospital data privacy and external research insights with distributed analytics

**DOI:** 10.1186/s12911-024-02549-5

**Published:** 2024-06-17

**Authors:** Benedetta Gottardelli, Roberto Gatta, Leonardo Nucciarelli, Andrada Mihaela Tudor, Erica Tavazzi, Mauro Vallati, Stefania Orini, Nicoletta Di Giorgi, Andrea Damiani

**Affiliations:** 1 Department of Diagnostic Imaging, Oncological Radiotherapy and Hematology, Università Cattolica del Sacro Cuore, Rome, Italy; 2 Department of Clinical and Experimental Sciences, Università degli Studi di Brescia, Brescia, Italy; 3 Department of Information Engineering, University of Padova, Padova, Italy; 4 School of Computing and Engineering, University of Huddersfield, Huddersfield, UK; 5 Alzheimer Operative Unit, IRCCS Istituto Centro San Giovanni di Dio Fatebenefratelli, Brescia, Italy; 6 Fondazione Policlinico Universitario Agostino Gemelli IRCCS, Rome, Italy

**Keywords:** GEN-RWD Sandbox, Privacy-preserving data sharing, Personalised medicine, Distributed analytics

## Abstract

**Background:**

Artificial intelligence (AI) has become a pivotal tool in advancing contemporary personalised medicine, with the goal of tailoring treatments to individual patient conditions. This has heightened the demand for access to diverse data from clinical practice and daily life for research, posing challenges due to the sensitive nature of medical information, including genetics and health conditions. Regulations like the Health Insurance Portability and Accountability Act (HIPAA) in the U.S. and the General Data Protection Regulation (GDPR) in Europe aim to strike a balance between data security, privacy, and the imperative for access.

**Results:**

We present the Gemelli Generator - Real World Data (GEN-RWD) Sandbox, a modular multi-agent platform designed for distributed analytics in healthcare. Its primary objective is to empower external researchers to leverage hospital data while upholding privacy and ownership, obviating the need for direct data sharing. Docker compatibility adds an extra layer of flexibility, and scalability is assured through modular design, facilitating combinations of Proxy and Processor modules with various graphical interfaces. Security and reliability are reinforced through components like Identity and Access Management (IAM) agent, and a Blockchain-based notarisation module. Certification processes verify the identities of information senders and receivers.

**Conclusions:**

The GEN-RWD Sandbox architecture achieves a good level of usability while ensuring a blend of flexibility, scalability, and security. Featuring a user-friendly graphical interface catering to diverse technical expertise, its external accessibility enables personnel outside the hospital to use the platform. Overall, the GEN-RWD Sandbox emerges as a comprehensive solution for healthcare distributed analytics, maintaining a delicate equilibrium between accessibility, scalability, and security.

## Background

Recently, artificial intelligence (AI) techniques have demonstrated their ability to enhance patient conditions and treatments, and are hence becoming one of the main tools for pursuing personalised medicine [1]. The underlying idea of personalised medicine is to find the most suitable treatment for patients by analysing heterogeneous data collected during clinical practice and patient’s daily life, with the clear results of driving the need of data availability for research purposes [2]. However, due to the sensitivity of patients’ personal information, data privacy has been a paramount concern in medical research over the past few years and it is one of the biggest challenges in personalised medicine. As a matter of fact, in the medical field, the risk does not only concern personal information such as names and contact information but also genetic data, medical conditions, and diseases related to the person. Therefore, it is not possible to share this kind of information freely among research institutions without considering the legal, ethical, and data ownership aspects involved. The Health Insurance Portability and Accountability Act (HIPAA) [3] in the United States and the General Data Protection Regulation (GDPR) [4] in Europe were drafted to balance the demand for security and privacy and the demand for access to data. Data-owning institutions, such as hospitals, are responsible for ensuring the privacy and security of data and are obligated to a strict collection and protection of information produced within their walls [5]. Given the resources invested in data collection, institutions tend to reject sharing their data, even in cases allowed by regulations, to avoid losing control over their archives [6]. This conflicts with the objectives of contemporary health research, which seek to extract valuable insights from the vast volumes of data gathered during clinical practice.

Several emerging technologies and approaches have been proposed to facilitate the privacy-preserving utilisation of sensitive data for collaborative research. These include data anonymisation [7], differential privacy [8], homomorphic encryption [9], Secure Multiparty Computation (SMC) [10], blockchain-based approaches [11] and Federated Learning (FL) or Distributed Analytics (DA) [12]. Among these, DA has gained significant popularity as it allows data to remain localised and distributed, minimising the risk of data breaches. With DA, raw data is not shared, reducing the exposure and potential risks for sensitive information. Moreover, it respects data ownership, allowing entities to retain control over their data while participating in collaborative research. Further, it is well-aligned with data protection regulations like GDPR, by minimising the transfer and processing of personally-identifiable information. This eliminates the need for data anonymisation or perturbation as in differential privacy approaches to protect patient privacy since the data itself is not shared. In certain cases, when data have bigger size, distributed analytics is preferred over computationally intensive techniques such as homomorphic encryption [13] and secure multiparty computation [14].

Since 2008, numerous proposals have emerged in terms of distributed analytics platforms, aiming to facilitate privacy-preserving data sharing. Among these, platforms like SHRINE/i2b2 [15], Clinerion [16], and TriNetX [17] provide software with a user interface accessible to researchers for operating queries or simple analysis on distributed datasets. In 2010, the first version of dataSHIELD [18] was introduced, enabling distributed analyses using the R statistical computing environment. In 2017, the Personal Health Train (PHT) framework was proposed, conceptualising a container-based data sharing infrastructure utilising a train analogy, where data sources are train stations and analysis methods are trains. PHT offers a generic solution for implementing various meta-analysis approaches. Examples of solutions developed within this framework are Vantage6 [19] and Welten et al. [20].

This paper aims to presents a solution in order to bridge the gap between hospital data privacy and the data availability requirements for clinical research: the GEmelli geNerator - Real World Data (GEN-RWD) Sandbox. We designed an architecture proposal for a distributed analysis platform that serves as a research playground for any stakeholder involved in medical research, from non-technicians to programmers, such as clinical researchers, policy makers, and pharmaceuticals [21]. The platform enables researchers to analyse clinical data in a distributed fashion without the need for data transfer between institutions. The sandbox derives its name from the secure and confined environment provided by the proposed infrastructure, where authorised users can freely explore and experiment with the data without gaining possession of it. The proposed platform is designed to be a modular multi-agent system capable of running any type of analysis pipeline which can be used by non-expert users to independently perform a diverse range of analyses without the supervision of a technical expert profile thanks to a user friendly web graphical user interface (GUI).

After this Background section, the article is organized as follows: Implementation section offers a thorough explanation of how the platform is implemented, focusing on each individual component. Discussion section introduces the GEN-RWD Sandbox’s functionalities and usage via its graphical user interface (GUI), highlighting its applications and unique features compared to other solutions, as well as addressing any limitations and suggesting future directions. Lastly, Conclusions section outlines the key points of the work and draws conclusions.

## Implementation

The basic functioning of GEN-RWD Sandbox is that authorised external users submit their research requests through a Graphical User Interface (GUI), which are then processed within the hospital. Only the results – from which it is not possible to trace back to patient-level original data – are shared with the external users outside the hospital’s domain. We followed as a design requirement the principle that the platform, in addition to offering a graphical tool for submitting requests for data analysis, should constitute a distributed multi-agent system that would guarantee high scalability and usability.

To achieve this, we propose a modular architecture where the GUI module is independent from the others and can be installed in any internet domain. Furthermore, to maximise flexibility, we divided the task of handling the processing of requests from the GUI into two separate modules, thus decoupling the complexity associated with interacting with the GUI and the security components from the execution of requests.

An overview of the communication protocol when a user submits an analysis request is given at the end of this section.

### General overview

**Fig. 1:**
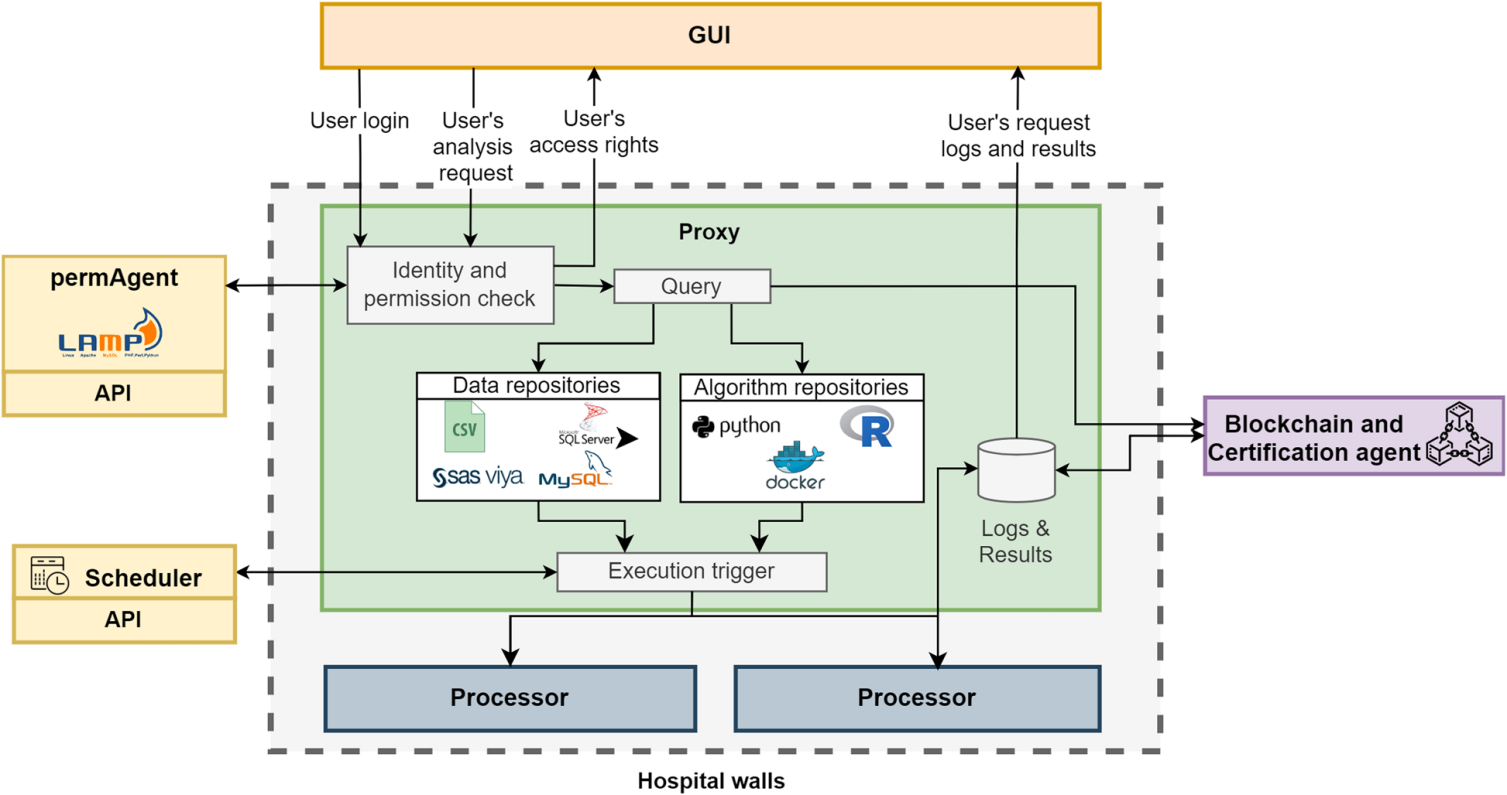
Overview of the GEN-RWD Sandbox’s modular architecture and functioning

The GEN-RWD Sandbox consists of two types of agents: operational modules and privacy and security components. The Processor, Proxy, Scheduler and GUI manage the operations of the platform, while the permAgent and Blockchain and Certification Agent implement the identity and access management, as well as the notarisation and blockchain certification of the elements exchanged with the user for privacy and security purposes. The operational modules are purposefully crafted to oversee the functions of the distributed analytics platform. Privacy and security components have been designed for the GEN-RWD Platform but oversee functions that could be present in other software platforms. Figure 1 shows at high level the GEN-RWD Sandbox architecture and the general workflow.

A user accesses the platform via the *GUI* after entering his credentials, which are verified by the Proxy by means of the IAM module API (*permAgent* block). Only after the credentials are verified is the user enabled to submit his or her request for analysis. The request is processed by the *Proxy*, which queries the requested data and algorithm from the respective repositories and prepares the execution instructions for the *Processor* module that will perform the analysis calculation. The results and logs produced by the Processor module are picked up by the Proxy which certifies and notarizes them in blockchain via the *Blockchain and Certification agent* component and then forwards them to the GUI which makes them available to the user.

While all other modules can be located outside the hospital walls, Proxy and Processor module instances must be located within hospital premises as the two modules in charge of handling data.

### Operational modules

The Processor, Proxy, Scheduler and GUI are the operational modules. Each of them is designed to fulfil a single function. The GUI is responsible for managing user interaction, e.g., collection of requests and presentation of results. The Processor has the task of executing the requests made. The Scheduler has the task of managing the scheduled and periodic execution of requests. Finally, the Proxy is the operational component with the role of managing the cooperation of all other modules including privacy and security modules, as well as managing the retrieval and storage of data and algorithms.

#### Processor

**Fig. 2:**
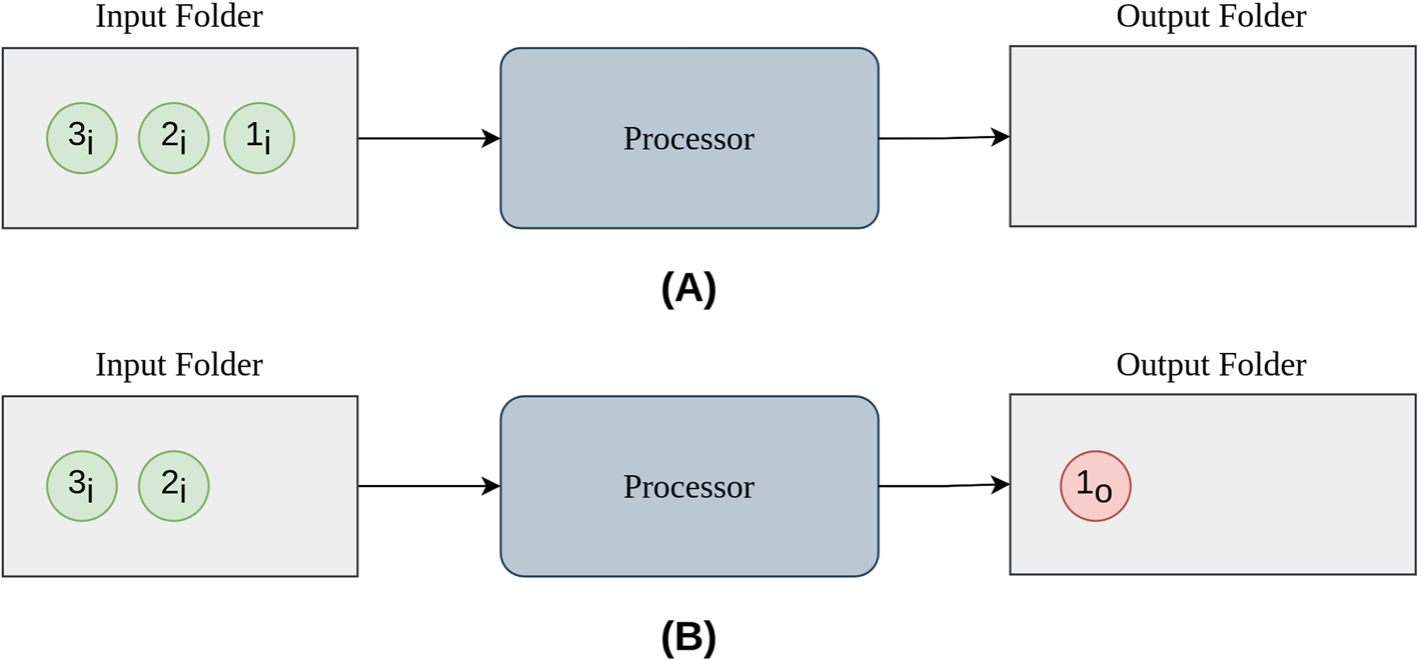
The Processor module’s core structure involves monitoring input folders. When a token is posted into one, it executes the script within (**A**) and reports the outcome to the assigned output folder as a new token (**B**)

The Processor module is the cornerstone component of the infrastructure, responsible for executing tasks according to a black box model that relies on a minimal structure (Fig. 2). This structure comprises a token, an input folder, the processing unit, and an output folder.

The token contains the details of the task to be executed and is designed as a collection of zipped files, including at least one XML file that characterises the task run and the structure of the token itself, referred to as a *descriptorXMLfile*. A token can contain both data and the script file, or information about their location within a local repository. The Processor is associated with one or more input folders and initiates the execution of a task when a token is inserted in one of them (Fig. 2A). While processed, the token is removed from the input folder. Upon completion of the computation, the Processor generates an output token, which is deposited into the output folder (Fig. 2B). Execution failures are handled by the Processor modules and all the related logs are forwarded to upper layers in the structure. This process is the core foundation of the infrastructure and is essential to the successful execution of any task or computation within the system.

The Processor can execute scripts written in R or Python or, more in general, any executable script encapsulated within a Docker image. Moreover, the Processor is able to process tasks in parallel launching each execution in background and keeping track to each one’s log. The Processor module was developed in R and encapsulated into the GSProcessor R package available at https://gitlab.com/benedetta.gottardelli/GSProcessor.git.

#### Proxy

The Proxy module, developed in Python (v3.9), is in charge of managing the cooperation of all modules of the infrastructure, as well as the retrieval and storage of data and algorithms.

The Proxy is the module of the architecture that guarantees data security by design, as it centralises all communications. The Proxy allows the decoupling between the outside world where the GUI is located and the hospital’s data archives, while the processor only takes care of executing the designated tasks within the hospital premises, and the GUI handles the interaction with the client.

In the proposed architecture, the Proxy module handles the function of retrieving and storing data and algorithms, and these data and algorithms managed by the Proxy module are considered certified by the infrastructure manager. Data can be stored within the Proxy as CSV files or retrieved on demand from external databases. In the current version, the Proxy is compatible with mySQL, SQLServer and SAS Viya environment. The algorithms are stored as Docker images in the local repository. The pulling of images from external repositories is not enabled, so that the functionality of the algorithms is tested and certified by the Proxy module manager, i.e. the data provider.

In addition, the Proxy module handles communication with all other modules by fulfilling the following additional functions:

1. Authenticating users by interrogating the identity and access manager module, the permAgent;
2. Monitoring for new tasks to be executed, starting the process by inputting the relevant tokens to the Processor or the Scheduler module if scheduling is requested;
3. Monitoring the output folder of the Processor to check if a result has been produced and forwarding any result item to the GUI;
4. Forwarding task requests and results to the Blockchain and Certification agent module.

Table 1 displays the general interaction scheme of each module with the Proxy in order for the platform to fulfill its basic functionalities. The Proxy represents the central node coordinating the communication workflow.

**Table 1:** Interactions of each GEN-RWD Sandbox module with the Proxy

Module	Functionality	From Proxy	To Proxy
GUI	User Authentication	• Authentication Response	• Authentication Request and ACK^a^
	Job Submission	• Job’s queued ACK^a^	• Job Request
	Job Outputs’ Presentation	• Job’s logs and outputs	
	Platform monitoring	• Number of available processor info Response	• Number of Available Processors Info Request
		• Algorithm and Datamart Metadata Update Info Response	• Algorithm and Datamart Metadata Update Info Request
permAgent	User Authentication	• Credential Verification Request	• Credential Verification Response
	User Permission Verification	• Permission Verification Request	• Permission Verification Response
Scheduler	Job Scheduling	• Scheduling Request	• Periodic Task Trigger
B &C Agent^b^	Job Notarization	• Job’s Notarization Request	• Job’s Notarization Results
	Job’s Output Signature	• Digital Signature Request	• Digitally signed Outputs
Processor	Job Execution	• Job execution details	• Job’s logs and outputs

^a^ACK: Acknowledgement Message

^b^ B &C Agent: Blockchain and Certification Agent

The Proxy module communicates with the Processor via the filesystem. On the other hand, communications with the GUI are handled in pooling via HTTPS. The Scheduler module, the permAgent and the Blockchain and Certification agent module provide APIs with which the Proxy communicates with them.

Thanks to the modularity of the architecture, the Proxy module can forward requests received from the GUI to multiple Processors, while also assuming the role of a dispatcher for computational load (Fig. 1). The Proxy monitors the number of forwarded, completed, and ongoing requests for each instance of the Processor and is in charge of balancing the work load among Processor modules. At the same time, the Proxy module can be configured to receive instructions from multiple GUIs, to allow the use of customised GUIs for different users.

#### Graphical user interface (GUI)

The GUI module, in the GEN-RWD Sandbox framework, exclusively handles the visual aspects and user interaction. It has no direct access to clinical data, as its purpose is solely to enable users to submit analysis requests and to visualise results.

GEN-RWD comes with an embedded GUI, but each developer can independently design their own GUIs, using the portfolio of APIs through which the Proxy provides computation/controls services and delivers results. In any case, the nature of the services allows the GUI to select existing datamarts offered only in the ways provided by the proxy and pair them with compatible computation algorithms.

The communication between the Proxy and the GUI occurs asynchronously, through repeated calls from the proxy to the GUI. This enables the management of the entire communication (i) through a single port (e.g., port 80), and (ii) ensures complete control of the communication solely to the proxy. The GUI restricts itself to presenting its requests through specific XML files (Message Master Table, or MMT, and Message Master Details, or MMD). These requests are captured by the Proxy and processed only if they align with internal privacy and security policies. The communication between the Proxy and the GUI takes place over an authenticated communication channel between the two agents, with a maximum time duration determined by the Proxy. On this communication channel, the creation of an additional channel occurs, established through user authentication against configurable authentication features (e.g., LDAP, internal authentication, etc.). The security of these exchanges can be further enhanced by adopting an encrypted protocol (e.g., HTTPS).

The embedded GUI is designed for non technical users as it allows them to access a list of the available algorithms and datamarts and to compose the desired analytic task via simplified interactive selection. Datamart subcohorts can be selected by querying the datamart via an Entity-Relationship based query builder or via a specific query builder for datasets standardised according to the Observational Medical Outcomes Partnership (OMOP) Commond Data Model [22].

The architecture is also designed to allow a Proxy to simultaneously interface with multiple GUIs, and at the same time, each GUI can communicate with multiple proxies, enabling the creation of networks that can easily scale in size.

The embedded GUI that we present in this work was developed using PHP, Shiny, and Python.

#### Scheduler

The Scheduler is the infrastructure component that manages the timing of the execution of periodic requests sent by the user via the graphical interface. It is in charge of triggering the task execution at fixed time instants as requested by the user. GEN-RWD Sandbox’s users can interact with the task list stored in the scheduler using the GUI, through which they can send signals to remove tasks from the task list, add new ones or freeze the execution of a specific task.

### Privacy and security components

After presenting the operational modules, we can now turn our attention to the other class of components, i.e. the privacy and security ones. The permAgent and the Blockchain and Certification agent handle GEN-RWD Sandbox’s privacy and security aspects. The permAgent is the platform’s Identity and Access Manager (IAM), while the Blockchain and the Certification agent deals with notarisation and accountability of the exchanged content.

#### permAgent - identity and access manager

Within the hospital walls, the verification of authentication privileges, or more generally, access to resources, is managed by a dedicated agent: the *permAgent* (permission agent). Access to every object, requested by the user through the GUI or by the proxy itself, undergoes *permAgent*’s permission verification.

The use of a resource is only possible within an additional authentication framework established between the permAgent and the Proxy through the exchange of a specifically created ticket in a session. The permAgent oversees validity characteristics (such as temporal validity of the session, etc.) to enable easy revocation of access rights by directly acting on a single agent. The interaction between the proxy and the permAgent occurs through microservices, allowing for easy relocation of agents across different physical machines by an easy reconfiguration of the system.

In the current version, the permAgent associates generic resources called *item* to users, group of users or group-subgroup hierarchies. It also rules the authenticating criteria, permitting several logical profiles (e.g.: LDAP authentication profiles, local users, etc.) per physical user.

#### Blockchain and Certification agent

A mechanism has been implemented within the Sandbox infrastructure to store and certify study results and metadata, aiming to ensure the reproducibility and accountability of tasks performed, which are well-known challenges in the field [23]. The implemented mechanism can be logically divided into two parts and is developed as a modular and pluggable Python library.

The initial part focuses on securely storing metadata and study results in an immutable and fault-resistant manner, achieved through the utilisation of Blockchain technology [24]. The immutability is attributed to the consensus mechanism, preventing malicious nodes from tampering with data in the blockchain. Additionally, the distributed ledger nature of blockchain addresses the single-point-of-failure problem. This component initially notarises the input token for the processor, which is devoid of data, and subsequently notarises the results of the computations. In detail, the process begins by generating the token, as described in the Processor section above, with the relevant metadata. Subsequently, the token hash code is calculated, stored off-chain, and then notarised in the blockchain. Similarly, when the Processor generates results, their hash is calculated, saved off-chain, and notarised in the blockchain.

This first component addresses the issue of reproducibility by preserving all details encapsulated in the metadata and in the results. It also contributes to accountability by tracking the user responsible for creating a study, along with its timestamp, providing verifiable proof of existence.

**Fig. 3:**
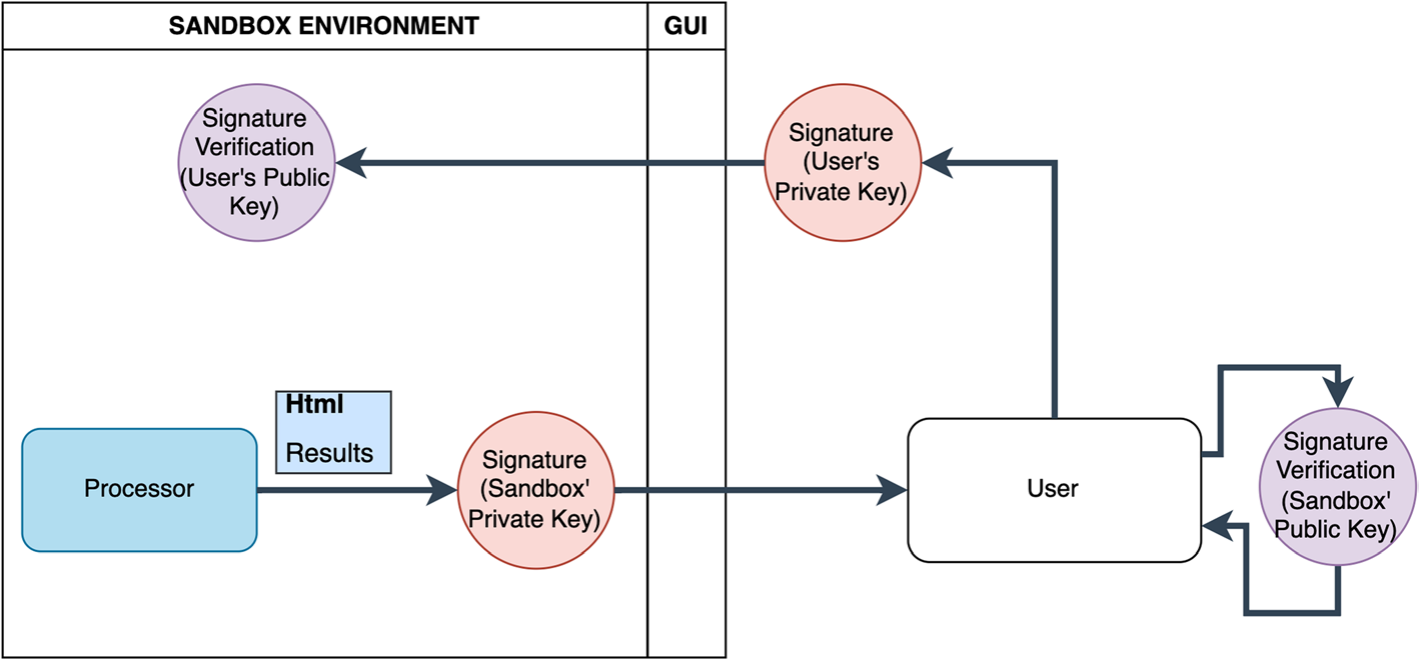
The digital signature workflow involves several actions. These include the initial signing action with the Sandbox’s private key, the initial user-side verification, signing with the user’s private key, and the subsequent double check within the GEN-RWD Sandbox

However, even though the sandbox records information about the studies, the accountability concern persists. For this reason, it is imperative for the user to directly assume responsibility for both the task and the generated results. Simultaneously, addressing a security challenge is essential, as users must have assurance that the results were generated by the sandbox and not by a malicious third-party actor. Hence, the second part of the module, represented in Fig. 3, involves employing a digital signature [25] to ensure accountability on both ends. The Sandbox infrastructure releases the report in a downloadable format, signed with its private key. Users can download the report and verify that the Sandbox generated the signature by using the Sandbox’s public key. At this stage, the report is not officially issued until the user reciprocally signs it with their private key, uploads it to the Sandbox infrastructure, and the user’s signature is authenticated using their public key. It is assumed that both the user and the infrastructure possess public keys that are, by definition, public and accessible to all.

The reader may have noticed that with the inclusion of this second stage, the total number of files pertaining to the results becomes three: the initial report, the one authenticated by the Sandbox, and the one authenticated by the users. It should be noted that all three reports are notarised both in the blockchain and off-chain.

The distributed ledger utilised in this paper is Hyperledger Fabric v2.4, enabling experimentation within a private environment. The chosen consensus algorithm is Raft [26]. We employed a configuration with a single node to maintain a lightweight and agile deployment.

### Communication flow

**Fig. 4:**
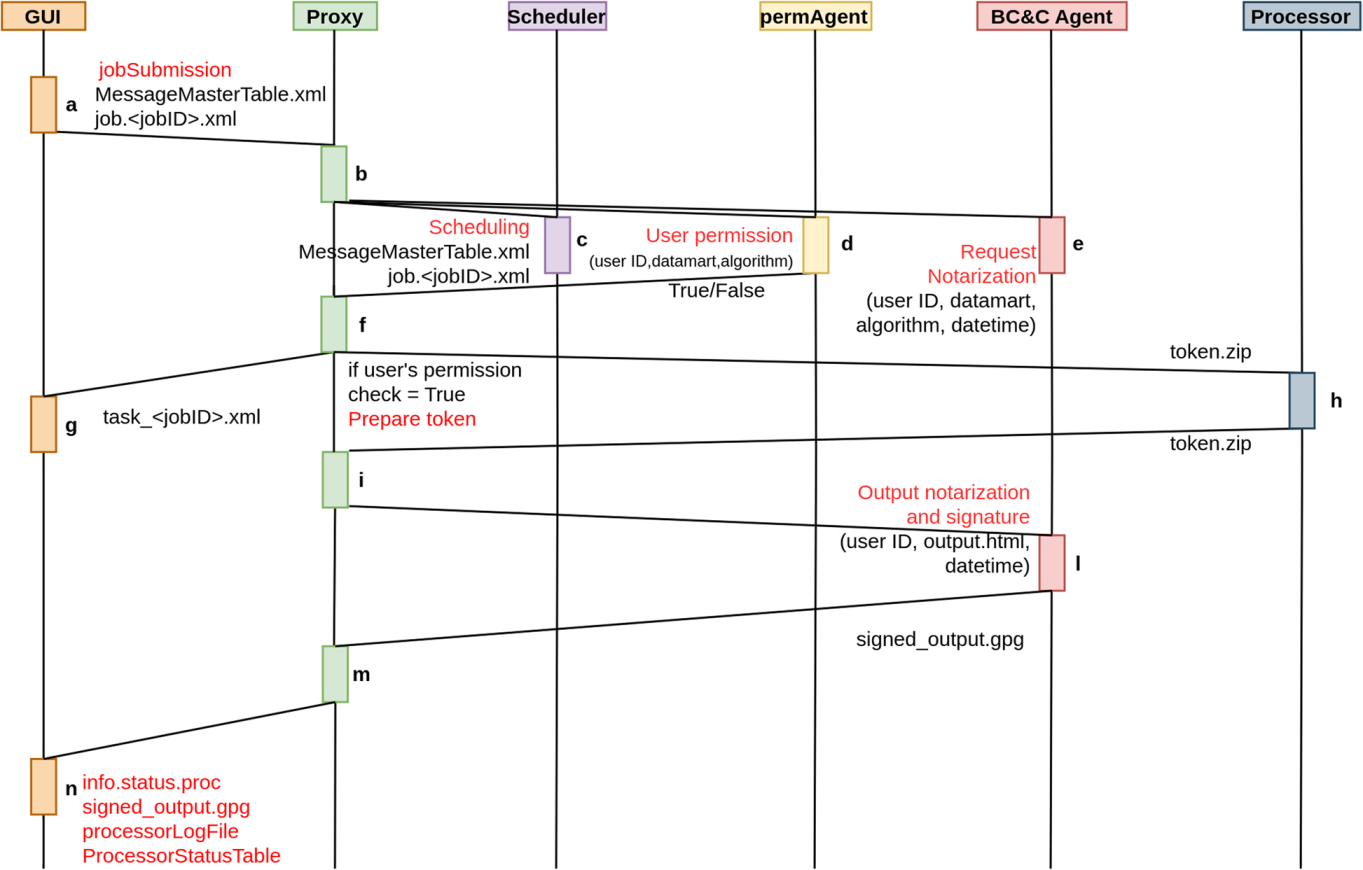
The communication flow that is triggered when a user submits a job via the GUI

Communication between the main modules of GEN-RWD Sandbox occurs asynchronously, with the Proxy module as manager. The proxy is always waiting for new submissions from the GUI or the Scheduler and constantly monitoring the processor’s folders for new logs or results. In turn, the Processor continuously monitors its input folder to detect the presence of instructions forwarded by the Proxy. Being on the same computer network within the hospital premises, the Proxy and the Processor communicate asynchronously through the filesystem. However, since the GUI is located on the internet outside the hospital’s computer perimeter, the proxy communicates with it through pool requests via HTTPS on port 80. Communication with all the other modules, i.e. permAgent, Blockchain and Certification agent and Scheduler, is handled at request with APIs. Figure 4 shows an example of communication flow triggered when a job is submitted by an authenticated user. The communication flow that is triggered when a user submits a job via the GUI:

- The GUI updates the MessageMasterTable.xml, which maintains a comprehensive list of all submitted jobs, and generates a file detailing the characteristics of each job.
- The Proxy monitors the associated GUI URLs and reads the details from the job_jobID.xml file.
- If scheduling was selected by the user, the Proxy forwards the obtained info to the Scheduler agent.
- The Proxy interrogates the permAgent IDM to check if the analysis pair *(datamart, algorithm)* can be used by the user.
- The Proxy forwards (*userID, datamart, algorithm, time stamp*) to the Blockchain and Certification Agent (BC &C Agent) in order to notarize the user’s request.
- If the permission check was successful, the Proxy prepares the token (*token.zip*) for the Processor based on the information received from the GUI and deposits it in the designated Processor’s input folder for computation.
- The Proxy sends log messages to the GUI, providing information about the selected processor for task execution coded as* info.assigned.proc* and the job’s status coded as *info.status.job*.
- The Processor triggers the task execution as soon as the token.zip file is made available in the input folder.
- The Proxy monitors the output and sync folders of the Processor.
- The Output token is notarised with blockchain and the output report is signed by the B &C Agent.
- The Proxy forwards B &C agent’s outputs to the GUI.
- The GUI processes the messages from the Proxy regarding the processor’s status and job’s outputs.

## Discussion

In this paper, we presented the modular architecture of a Distributed Analytics platform that is user-friendly and accessible to any kind of user including non-technical ones, the Gemelli Generator - Real World Data (GEN-RWD) Sandbox. Our main objective is to provide stakeholders engaged in clinical research, particularly in the field of personalised medicine, which requires substantial amounts of data-driven evidences, with direct access to the clinical patient-level data collected by hospitals.

**Fig. 5:**
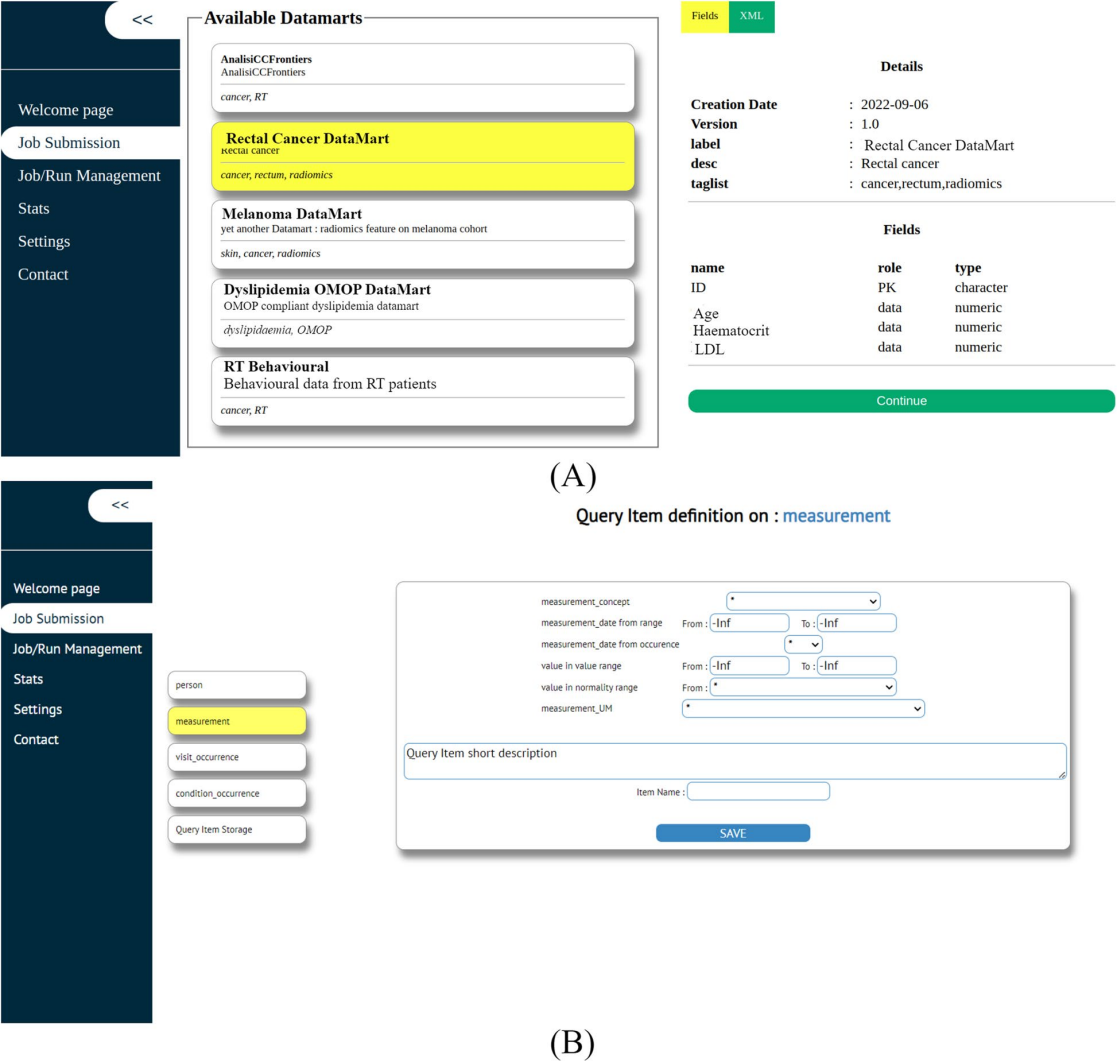
GEN-RWD Sandbox’s GUI Job Submission page - Datamart selection tab (**A**) and OMOP query builder tab (**B**)

Users of the GEN-RWD Sandbox can access the platform by authenticating themselves via the login prompt accessible via an Internet browser. On the Job Submission page, users have the capability to create their analysis requests by choosing a datamart-algorithm pair from the available lists of items. Figure 5A shows an example where the “Rectal Cancer DataMart” is selected and its characteristics are displayed to the user in the right section of the page, similarly algorithms can be selected in this GUI section. The OMOP-compliant datamarts can be queried using the embedded query builder within the GUI, as depicted in the Fig. 5B.

**Fig. 6:**
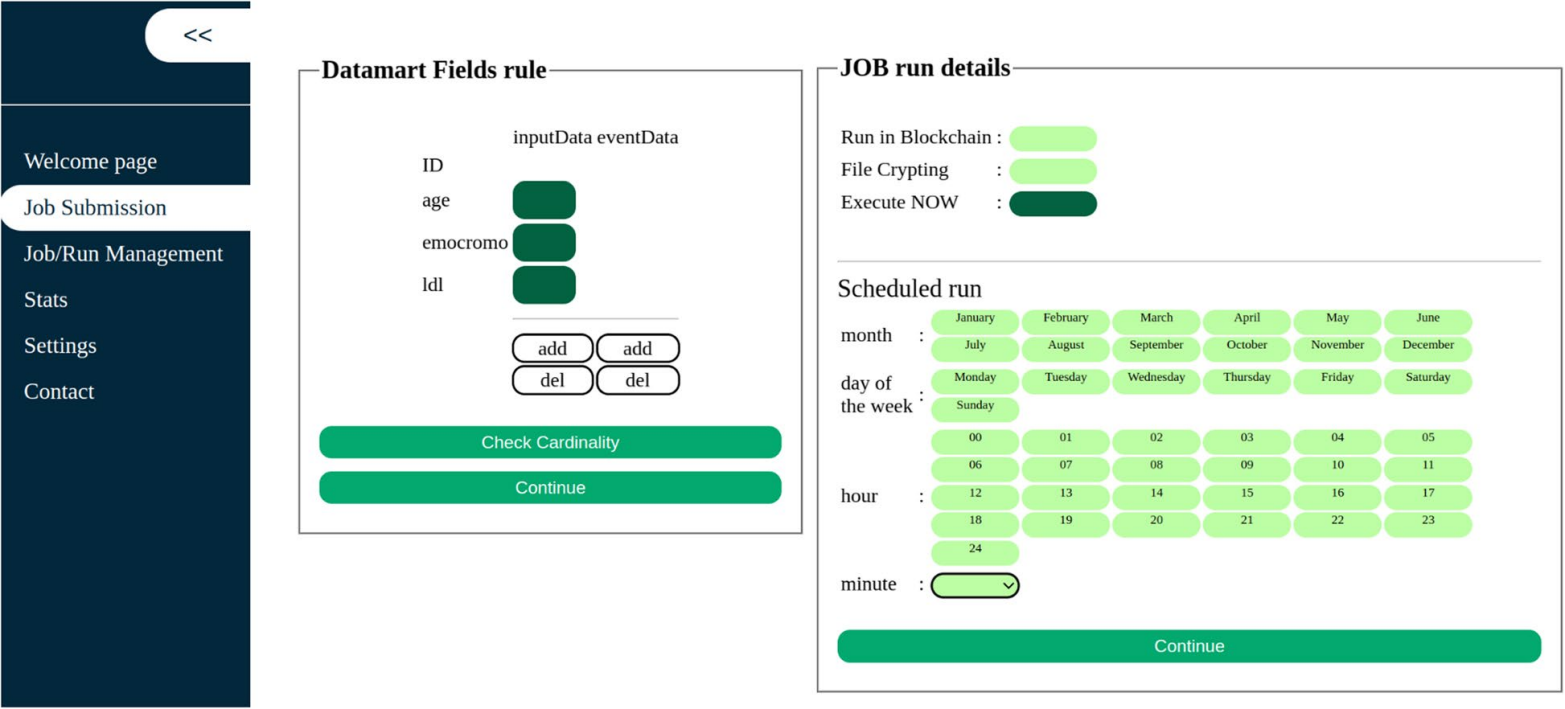
GEN-RWD Sandbox’s GUI Job Submission page - Job settings tab

On the Job Submission page, users can specify which datamart fields are intended for use as algorithm inputs or targets and configure the Job run settings (Fig. 6).

**Fig. 7:**
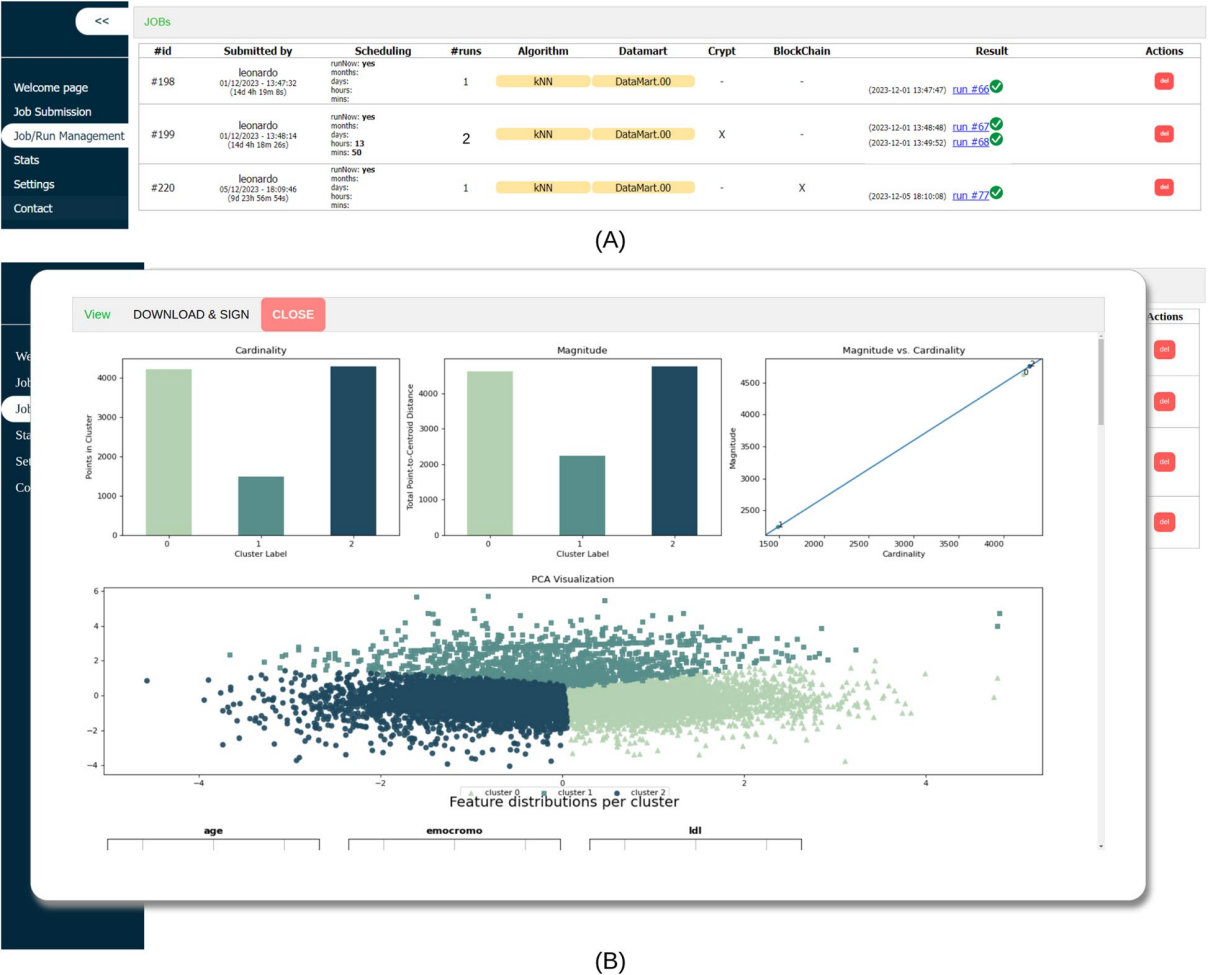
GEN-RWD Sandbox’s GUI Job/Run Management page - Job list (**A**) and Job’s result page (**B**)

Additionally, users can enable blockchain notarisation and result encryption/signature on this page. Simultaneously, users have the option to either execute the job immediately or schedule it for a later time. After submission, the Proxy manages and the Processor executes the Job. Users monitor the status and results of their Jobs from the Job/Run Management page (Fig. 7A). Finally, access the job results by clicking on the hyperlink in the Result column, the result of the work can be accessed (Fig. 7B).

The GEN-RWD Sandbox is designed in a modular fashion, allowing for the independent development, testing, and deployment of individual components. This modular approach enhances maintainability, facilitates updates, and enables a high degree of flexibility in adapting to evolving requirements. Scalability is a key strength of the GEN-RWD Sandbox, ensuring that it can handle increasing workloads efficiently. The architecture is designed to seamlessly scale both vertically and horizontally, making it suitable for a broad range of applications. A distinguishing feature of the software infrastructure is its adaptability to incorporate new functionalities. The modular design allows for the easy integration of additional features or updates without disrupting the overall system, ensuring longevity and relevance in a rapidly evolving technological landscape. Personalised GUIs can be designed according to users’ needs, as long as they support the implemented communication protocol with the Proxy module. In addition, some of the developed modules that make up the platform can also be used outside the GEN-RWD Sandbox ecosystem. The Blockchain and Certification agent constitutes a notarisation and digital signature agent that can also be used to certify the communication exchanges in other settings. Similarly, the Processor module, which basically constitutes an executor of requests in the form of tokens, can be used to automate other types of processes, such as extraction, transformation and load (ETL) processes. The GEN-RWD Sandbox integrates a diverse set of technology frameworks, including Docker for containerisation, Blockchain for secure and transparent transactions, digital signature for authentication and data integrity, OMOP for standardised healthcare data representation, relational databases for structured data storage, R and Python for both analytics and agent development, API for seamless connectivity, PHP for web application development, and Shiny for interactive and user-friendly visualisations.

One of the architectural choices that guided the development of this platform is related to the need to allow users outside the hospital domain to perform analysis and learning on the data. For this reason, we realised the decoupling between the GUI and the data management modules. In this setting, the GUI can be located anywhere on the Internet and is therefore accessible externally, ensuring widespread usability. This feature enables users to interact with the system from various locations, promoting collaboration and accessibility. This choice required us to design asynchronous pulling communication from the inside, i.e., from the Proxy, to the GUI in order to guarantee the security of communication against the interference of malicious agents inside the infrastructure in the data domain area. The other design choice was to create a user-friendly interface in order to further extend the usability of the platform, but at the same time adjust the degree of freedom of interaction. The data and algorithms on which it is possible to carry out analyses from the platform are proposed by the Sandbox manager or by the data provider itself and are therefore considered certified and of possible use, i.e. not harmful. It should be noted that there are some aspects relating to data management and trustworthiness of results that are left entirely to the GEN-RWD Sandbox’s manager. Sandbox is, in fact, agnostic with respect to the data and tasks provided. This implies that aspects such as preprocessing, data cleaning, and the identification/management of possible biases in the provided information - which are a frequent issue in real data analysis - are not handled automatically by the platform, but remain the responsibility of the algorithm and data provider. In future work, we plan to elaborate on these aspects in the practical use of GEN-RWD Sandbox for actual clinical research. Moreover, what the user is allowed to interact with is managed by an access management system also controlled by the infrastructure manager.

In the infrastructure design, we have incorporated a certification agent to establish a mechanism for notarising the analyses conducted and certifying both the authenticity of the content received and its successful fruition by the user. This ensures the provision of immutable guarantees on the analyses carried out for both parties and thus trust in the platform on the part of both the user and the data provider.

The usability of other platforms such as SHRINE/i2b2 [15], Clinerion [16], and TriNetX [17] falls short compared to the GEN-RWD Sandbox, as they permit only basic data operations, such as counts, survival analysis, and logistic regression. Additionally, solutions like those from Clinerion and TriNetX are proprietary products developed by hospital networks, lacking the openness of an open-source system, such as GEN-RWD Sandbox. Despite R’s reputation as a powerful tool for statistical analysis, DataSHIELD [27] faces limitations when training complex deep learning models. This restriction is attributed to R’s toolbox, which encompasses a confined set of functions and methods, limiting its capabilities in this specific domain. The GEN-RWD Sandbox addresses potential language-specific issues by ensuring compatibility with Docker containers, thereby offering flexibility for performing a wide range of analytical tasks. While lacking a user interface geared towards non-technical IT personnel, the distributed analytics infrastructures based on Personal Health Train (PHT) [28], exemplified by Vantage6 platform [19] and Welten et al. [20], exhibit exceptional flexibility and usability. This is attributed to their utilisation of Docker technology, employing containerisation to disseminate algorithms for application on data across participating institutions. In contrast to these infrastructures, the proposed solution features algorithms sourced from local repositories rather than distributed ones. Consequently, the control and functionality of the algorithm reside with the data provider. Moreover, in the GEN-RWD Sandbox, the authority to execute a specific algorithm on its respective dataset is vested in the data provider, i.e., the infrastructure manager, ensuring the desired levels of flexibility.

Currently, the main limitation of our platform is that it facilitates the learning of models in a distributed mode exclusively within a single institution. Indeed, the primary objective was to create an infrastructure that would allow controlled access to hospital data. However, due to the considerable modularity of the proposed architecture, we aspire to rearrange the functionalities of specific modules. This reorganisation aims to extend the learning capability of the model to a network of hospitals, with each hospital installing modules designated by the GEN-RWD Sandbox in its own premises.

Even if the feedback of our data analysts were positive in terms of data security and privacy, flexibility, scalability, and reliability requirements, an extensive usability test on the entire architecture is needed. For this reason we are currently working on the definition of a user-machine interaction evaluation framework which could be helpful to frame the general and specific users’ needs and provide metrics to compare this kind of architecture.

We are aware of the risks associated with differential privacy when granting users the ability to query data. Excessively specific conditions pose a potential threat of identifying individual patient data, thereby compromising the secure data aspects outlined in the security assessment framework, the Five Safes, for distributed analytics platforms [29, 30]. Simultaneously, there is the concern that a malicious user may intentionally seek to extract patient-level data from aggregated results by repetitively executing queries through reverse engineering operations [31]. To address these challenges, we are committed to integrating a Differential Privacy module within our platform in the near future [32]. This module would aim to introduce noise into the data extracted from queries and to limit user’s query possibilities, preventing the identification of patient data from aggregated results.

While the GEN-RWD Sandbox could already be used as a platform for distributed analytics in hospital settings, as further future works, we plan to enhance the current version of GUI to further facilitate user analysis queries. We believe that providing information on the compatibility between algorithms and proposed datasets, as well as the types of columns that can be used for each algorithm (e.g., binary outcome for logistic regression), will be crucial for the future utilization of the platform. We also plan to develop a principled methodology for adding new algorithms to our Sandbox analysis catalog and one specific for developing customized GUI compatible with the GEN-RWD Proxy module, in order to further expand the Sandbox capabilities and usability.

## Conclusions

In this paper, we introduced the Gemelli Generator - Real World Data (GEN-RWD) Sandbox, a modular multi-agent platform designed for distributed analytics in the healthcare domain. The primary objective of this infrastructure is to facilitate the utilisation of hospital data by external researchers while maintaining privacy and ownership by avoiding the need for data sharing.

The architectural framework was developed with the goal of enhancing usability compared to previously proposed solutions in the literature, all the while upholding a high degree of flexibility, scalability, and security. Through a user-friendly graphical user interface (GUI), the GEN-RWD Sandbox caters to users of all skill levels, including those lacking information technology (IT) proficiency. Additionally, the externalisation of the GUI from the hospital perimeter allows personnel outside the hospital to access the platform. Docker compatibility adds an extra layer of flexibility to the platform. The scalability of the platform is ensured through the modularity of the infrastructure, allowing the combination of the Proxy module with multiple Processor modules (i.e., computing modules) and various GUIs. To guarantee the security and trustworthiness of the infrastructure, the IAM component, permAgent, and a module for immutable notarisation in Blockchain, along with the certification of the identity of information senders and receivers, are incorporated.

## Data Availability

• Project name: GEN-RWD Sandbox Processor (GSProcessor) • Project home page: https://gitlab.com/benedetta.gottardelli/GSProcessor • Operating system(s): Linux OS • Programming language: R • Other requirements: Docker • License: LGPL-3.0 • Any restrictions to use by non-academics: no restrictions, please attribute work by citing this paper.

## Abbreviations

AI: Artificial Intelligence
ACK: Acknowledgment
DA: Distributed Analytics
ETL: Extraction, Transformation and Load
FL: Federated Learning
GDPR: General Data Protection Regulation
GEN-RWD: GEmelli geNerator - Real World Data
GUI: Graphical User Interface
HIPAA: Health Insurance Portability and Accountability Act
IAM: Identity and Access Manager
IT: Information Technology
PHT: Personal Health Train
SMC: Secure Multiparty Computation
